# Hemoglobin Levels Modulate Nitrite Toxicity to *Daphnia magna*

**DOI:** 10.1038/s41598-018-24087-7

**Published:** 2018-05-08

**Authors:** Stephanie A. Eytcheson, Gerald A. LeBlanc

**Affiliations:** Department of Biological Sciences North Carolina State University, Raleigh, NC 27695 USA

## Abstract

Nitrogenous compounds enter the environment through various anthropogenic sources. Among these are nitrate (NO_3_^−^) and nitrite (NO_2_^−^) which can oxidize the heme moiety of hemoglobin and reduce the oxygen-carrying capacity of the molecule resulting in toxicity. Of the two anions, nitrite is more toxic. Hemoglobin levels are influenced by environmental conditions; thus, we hypothesized that hemoglobin levels would influence the toxicity of nitrite with low hemoglobin levels resulting in enhanced toxicity and high hemoglobin levels resulting in reduced toxicity. We tested this hypothesis by elevating hemoglobin levels with pyriproxyfen treatment and lowering hemoglobin levels using siRNA in *Daphnia magna*. Exposure to pyriproxyfen significantly elevated hemoglobin mRNA levels and induced copper coloration of the organisms, indicative of increased hemoglobin protein accumulation. siRNA treatment significantly reduced hemoglobin mRNA levels in both untreated and pyriproxyfen-treated organisms and attenuated copper coloration. Pyriproxyfen treatment increased the tolerance of daphnids to the acute toxicity of nitrite approximately 2-fold while siRNA treatment significantly decreased the tolerance of daphnids to nitrite toxicity. Results indicate that increased hemoglobin levels increase the tolerance of daphnids to nitrite toxicity which may serve to protect daphnids in environments subject to hemoglobin-elevating hypoxia or elevated temperatures.

## Introduction

Seventy-eight percent of the earth’s atmosphere is comprised of nitrogen, most of which is biologically unavailable due to the inability of most organisms to break the bonds of N_2_^1^. Atmospheric nitrogen is processed into biologically available forms by nitrifying bacteria. These bacteria “fix” atmospheric nitrogen to NH_4_^+^ which can be oxidized to NO_2_^−^ and then NO_3_^−^. Levels of nitrite and nitrate in the environment are typically regulated through assimilation by plants or through conversion back to atmospheric nitrogen by denitrifying bacteria^2^. The US Environmental Protection Agency has set the drinking water standard for nitrate at 10 mg N/L (about 45 mg nitrate/L) and nitrite at 1 mg N/L (about 3 mg nitrite/L)^3^ while the World Health Organization has set limits at 50 mg/L for nitrate and 3 mg/L nitrite; these limits are set for short-term exposures^4^. Though limits have been set to protect sensitive subpopulations from the acute toxic effects of nitrate and nitrite, contamination with these nitrogen oxides remains problematic with levels in freshwaters often exceeding allowable limits^5^. Nitrogenous compounds are introduced into the environment by various anthropogenic activities such as the use of concentrated animal feeding operations, nitrogen-based fertilizers, and the burning of fossil fuels. These activities have altered the nitrogen cycle resulting in increased levels of biologically available nitrate and nitrite^6,7^. Increasing levels of these biologically available nitrogen oxides have consequences both environmentally (harmful algal blooms, hypoxic waters, ocean acidification) and on human health (methemoglobinemia, birth defects, cancer.)^5,6^.

One potential mechanism of nitrite toxicity is oxidation from the ferrous to the ferric state of the heme moiety of hemoglobin^8,9^. Hemoglobin or hemoglobin-like molecules are found in all kingdoms of organisms, from Archaea and Bacteria to Plantae and Animalia^10–13^. Hemoglobin levels are often regulated by environmental factors. For example, hemoglobin synthesis can be induced by hypoxia or elevated temperature^14,15^. Hemoglobin functions primarily in two important ways: 1) to sequester and deliver oxygen to the organism and 2) to transport other gaseous signaling molecules^16^. Hemoglobin is able to transport such molecules via reversible binding to iron which is coordinately bound to a protoporphyrin IX (heme group)^17^. Oxygen is able to bind to the ferrous form of iron (Fe^2+^) in the heme group^18^. Exposure to nitrite results in oxidation of the ferrous ion into the ferric ion (Fe^3+^) creating methemoglobin to which oxygen is unable to bind^19,20^.

We tested the hypothesis that hemoglobin levels modulate the tolerance of *D*. *magna* to nitrite toxicity. We proposed that elevated hemoglobin levels would offset the oxidation of heme moieties by nitrite thus leaving sufficient unaltered hemoglobin to support normal function. Conversely, we proposed that reduced hemoglobin levels would reduce the tolerance of daphnids to the toxicity of nitrite by providing insufficient hemoglobin reserves for the transport of oxygen. Here, we increased hemoglobin levels in daphnids by treatment with the insecticide pyriproxyfen, which induces hemoglobin production via activation of the methyl farnesoate signaling pathway^21^, and decreased hemoglobin levels using siRNA.

## Results

### siRNA Optimization

**Figure 1:**
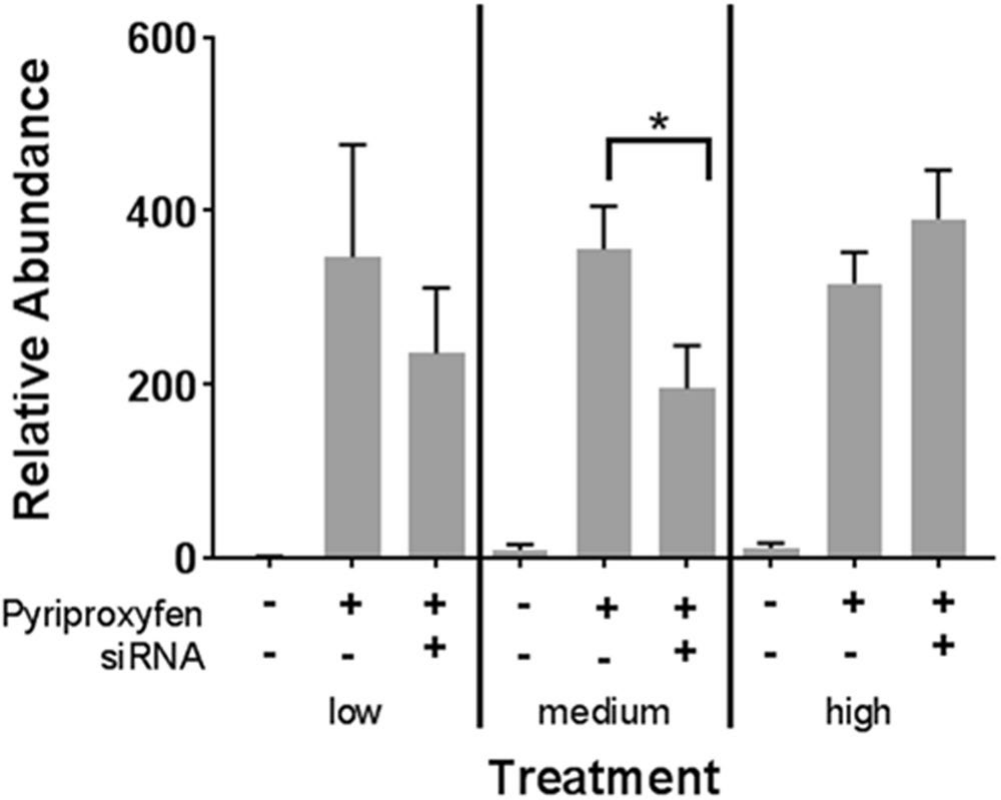
Relative abundance of Dhb2 mRNA in *D*. *magna* provided low (2.4 × 10^7^ cells/100 mL), medium (7.2 × 10^7^cells/100 mL), and high (12 × 10^7^ cells/mL) concentrations of bacterial cells containing the L4440 vector or L4440 vector expressing Dhb2 siRNA with or without pyriproxyfen treatment. Data are presented as the mean and standard deviation (n = 3). Results are normalized to the control group (no pyriproxyfen and siRNA) fed the low concentration of bacterial cells. An asterisk denotes a significant (*p* < 0.05) difference between the connected data (One-way Analysis of Variance, Tukey’s Multiple Comparisons test).

**Figure 2:**
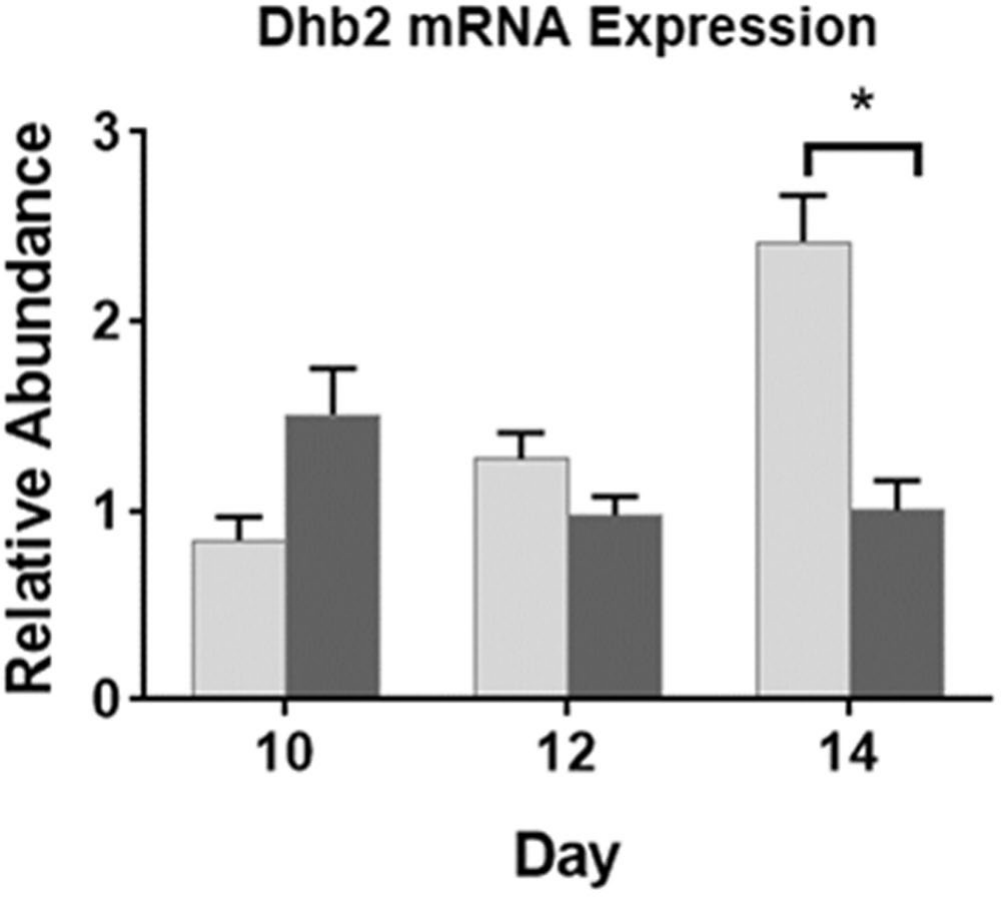
Time course for the reduction of Dhb2 mRNA levels in *D*. *magna* fed bacteria producing the dsRNA targeting Dhb2. Daphnids were fed bacteria containing either L4440 vector (light gray) or L4440 vector containing the Dhb2 siRNA (dark gray) for the indicated number of days then evaluated for Dhb2 mRNA abundance. Abundance of mRNA in organisms fed Dhb2 siRNA was normalized to mRNA levels in organisms fed empty vector for each day. An asterisk denotes a significant (*p* < 0.05) difference between the connected data (One-way Analysis of Variance, Bonferroni’s Multiple Comparisons test).

Exposure of daphnids to 3.0 nM pyriproxyfen significantly increased the level of hemoglobin Dhb2 mRNA (Fig. 1). Feeding organisms *E*. *coli* that expressed dsRNA to Dhb2 at 7.2 × 10^7^ cells/100 mL medium significantly (~50%, *p* < 0.05) reduced Dhb2 mRNA levels in pyriproxyfen-exposed daphnids. We next investigated the minimum amount of time of feeding dsRNA required to reduce Dhb2 mRNA levels in daphnids. Dhb2 mRNA levels were significantly reduced after fourteen days of feeding (*p* < 0.05, Fig. 2).

### Modulation of Hemoglobin

**Figure 3:**
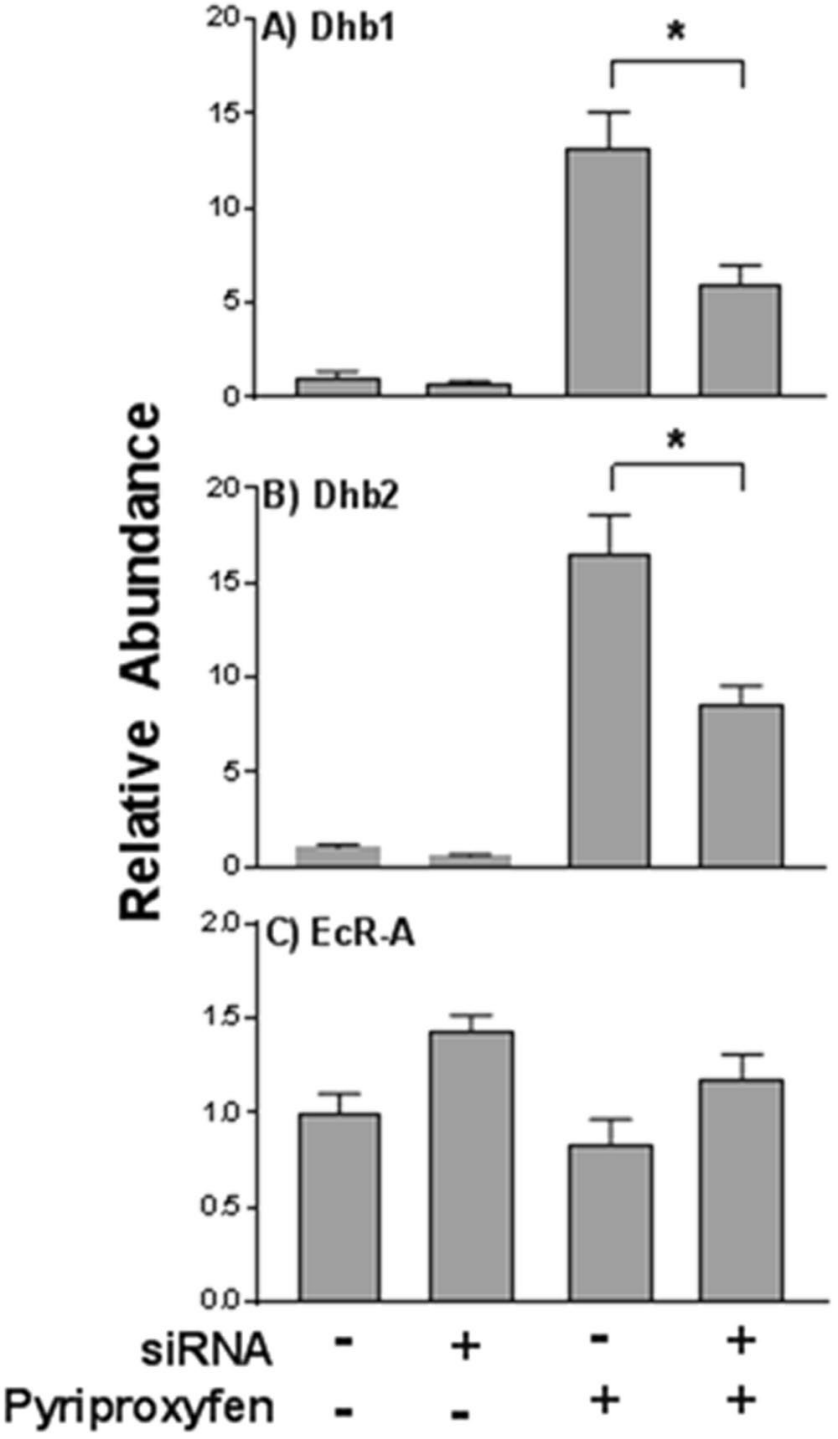
Relative abundance of Dhb1 (**A**), Dhb2 (**B**), and EcR-A (**C**) mRNA in *D*. *magna* provided L4440 vector or L4440 vector expressing Dhb2 siRNA with or without pyriproxyfen treatment. Data are presented as the mean and standard deviation (n = 3). Results for each gene are normalized to the control (no siRNA and pyriproxyfen). An asterisk denotes a significant (*p* < 0.05) difference between the connected data (One-way Analysis of Variance, Tukey’s Multiple Comparisons test).

We evaluated the specificity of hemoglobin induction by pyriproxyfen and suppression by siRNA by evaluating levels of Dhb1, Dhb2, and EcR-A (ecdysteroid receptor) mRNA following treatments. Daphnids were provided the vector-containing bacteria for 14 days then exposed to pyriproxyfen for 4 days (with continued provision of the bacteria). Pyriproxyfen (6.0 nM) significantly elevated Dhb1 and Dhb2 mRNA levels in daphnids fed bacterial cells containing empty vector (Fig. 3). This elevation was significantly (*p* < 0.05) attenuated among daphnids provided bacteria expressing dsRNA (Fig. 3). EcR-A mRNA levels were not modulated by pyriproxyfen or siRNA. Dhb1 and Dhb2 nucleotide sequences are highly similar and distinct from that of EcR-A. Results indicate that the Dhb2 dsRNA also annealed to Dhb1. Modulation of hemoglobin levels resulted in a phenotype wherein daphnids exposed to pyriproxyfen developed increased copper-coloration which was attenuated by feeding of dsRNA targeting hemoglobin.

### Modulation of Nitrite Toxicity by Pyriproxyfen and siRNA

**Figure 4:**
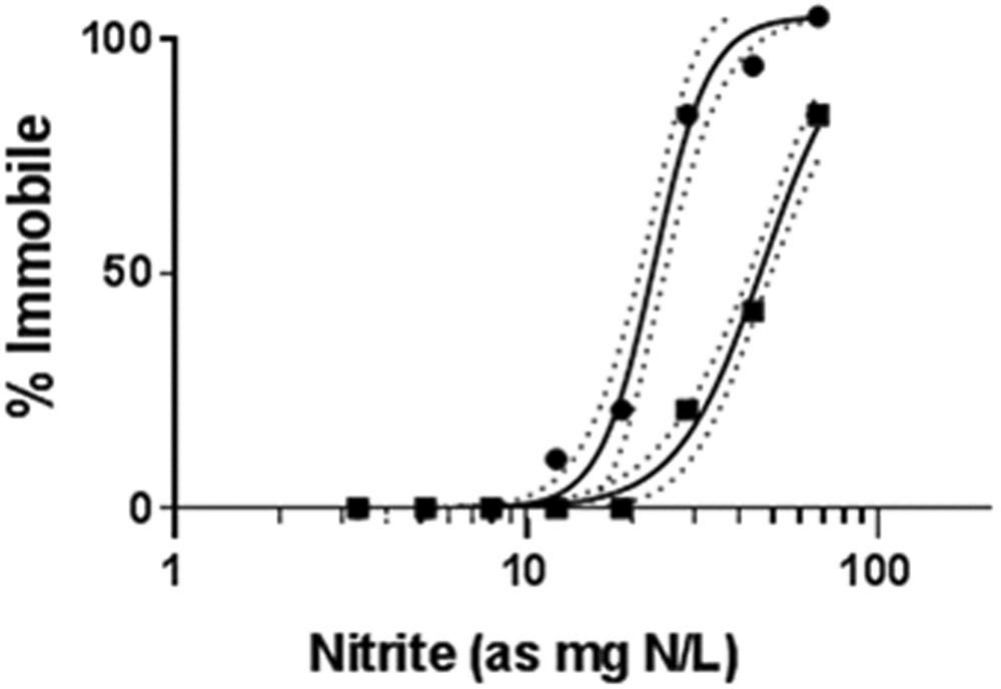
Concentration-response of *D*. *magna* reared in the absence (circles) and presence (squares) of 3.0 nM pyriproxyfen for 48 hours prior to exposure to various concentrations of sodium nitrite. Data points are presented as the percentage immobilized daphnids (n = 10) at each concentration of NaNO_2_. Confidence intervals (95%) are depicted by dotted lines.

The ability of pyriproxyfen to modulate the toxicity of nitrite was evaluated. We hypothesized that increased hemoglobin from pyriproxyfen treatment would reduce the ability of nitrite to elicit toxicity. Daphnids (5 days old) were exposed, or not (control), to 3.0 nM pyriproxyfen and then exposed to a concentration series of sodium nitrite. Mobility of individual daphnids was assessed after 48 hours of exposure to sodium nitrite. The 48-hour EC50 of nitrite with daphnids that were not exposed to pyriproxyfen was 23 mg N/L (95% confidence interval (CI): 21–25 mg N/L). The 48-hour EC50 for nitrite nearly doubled among daphnids pre-exposed to pyriproxyfen (55 mg N/L, 95% CI: 50–59 mg N/L). The no observed effect concentration (NOEC) for nitrite increased from 6.5 mg N/L to 18 mg N/L with pyriproxyfen exposure (Fig. 4). Results were consistent with the hypothesis that increased hemoglobin levels reduced the toxicity of nitrite. We next sought to further test the hypothesis by evaluating the toxicity of nitrite following the induction (pyriproxyfen treatment) and suppression (siRNA treatment) of hemoglobin.

**Figure 5:**
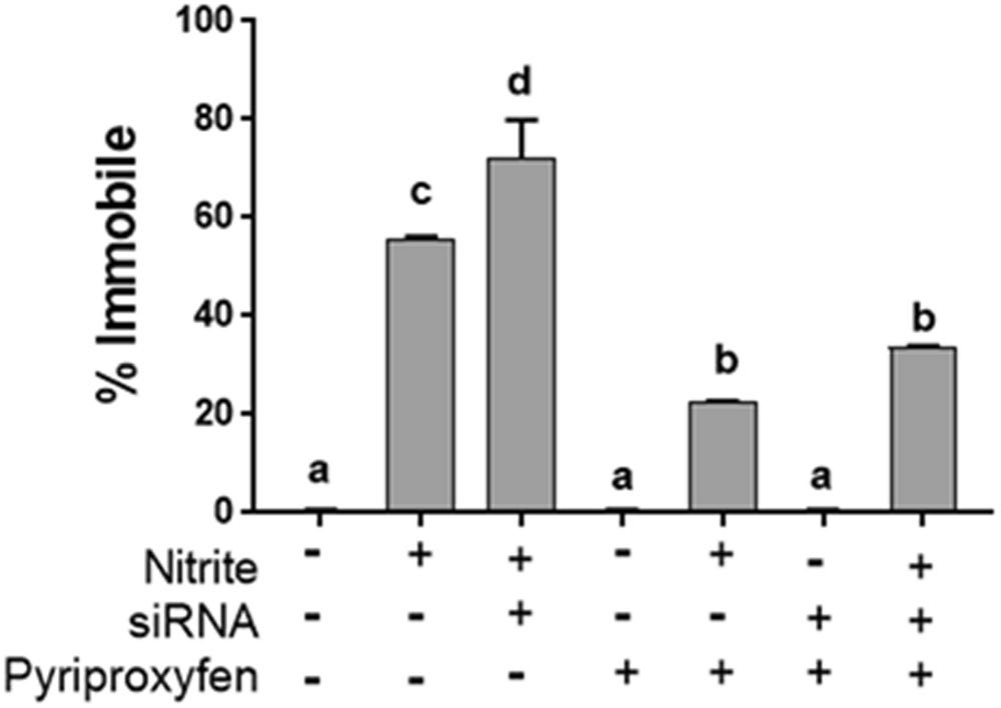
Immobilization of *D*. *magna* exposed to sodium nitrite following treatment with Dhb2 siRNA and/or pyriproxyfen. Data are presented as the percentage immobilized daphnids (9 daphnids per replicate, 2 replicates per treatment). Treatments denoted with different letters were significantly (*p* < 0.05) different (One-way Analysis of Variance, Tukey’s Multiple Comparisons Test).

We chose a concentration of sodium nitrite that would immobilize ~50% of control animals (i.e. ethanol control and fed bacteria containing empty vector) so as to detect either increased or decreased tolerance to nitrite. No toxicity occurred among control organisms or other groups not exposed to nitrite (Fig. 5). Nitrite exposure elicited ~50% immobility among control daphnids. dsRNA feeding alone resulted in a significant reduction in tolerance to nitrite toxicity (*p* = 0.0083). Pyriproxyfen treatment alone significantly increased tolerance to nitrite toxicity when compared to organisms not exposed to pyriproxyfen (*p* = 0.0001). Nitrite toxicity was significantly reduced among daphnids fed dsRNA and exposed to pyriproxyfen as well as nitrite when compared to organisms fed dsRNA and exposed to nitrite without pyriproxyfen exposure (from 70% to 30%, *p* < 0.0001). Overall, tolerance to the toxicity of nitrite was directly related to hemoglobin levels.

## Discussion

In the present study we hypothesized that hemoglobin levels modulate tolerance to nitrite toxicity in the model organism *D*. *magna*. Under all scenarios evaluated, tolerance of daphnids to nitrite toxicity tracked with hemoglobin levels. We propose that high hemoglobin levels allow for the oxidation of the heme moiety by nitrite to occur with sufficient unaltered hemoglobin remaining for normal oxygen transport. Conversely, we propose that low hemoglobin levels result in oxidation of heme groups to an extent which depresses oxygen provision to cells.

Hemoglobin levels were suppressed in this study using siRNA. The protocol used to knock down hemoglobin gene expression via feeding dsRNA was based upon that described by Schumpert, *et al*.^22^ for the suppression of phenoloxidase. In their study, smaller daphnid species were used (*D*. *pulex*, ≤ 3.0 mm, *D*. *pulicaria*, ≤ 3.0 mm, *and D*. *melanica*, ≤ 2.0 mm); thus, we optimized feeding for *D*. *magna* (≤5.0 mm)^23–25^. The lowest concentration of bacterial cells (2.4 × 10^7^cells/mL) used in this study was the optimal concentration used by Schumpert *et al*.^22^. With *D*. *magna*, maximum suppression of Dhb2 mRNA levels was observed at a concentration of 7.2 × 10^7^ cells/100 mL medium. At this concentration, we observed a 50% reduction in mRNA abundance. The reason for our inability to knock down more than 50% of the Dhb1 and Dhb2 mRNA is unknown but may be due to toxicity associated with feeding higher concentrations of *E*. *coli*. Overall performance of daphnids has been shown to decrease with increasing ratio of *E*. *coli*/algal cells in the medium^26^. Further, 12 × 10^7^ cells *E*. *coli*/100 mL medium was previously shown to be toxic to daphnids^22^. We suspect that food consumption by *D*. *magna* may have decreased with increasing *E*. *coli* concentration resulting in reduced dsRNA delivery and commensurate loss of the suppressive action of the siRNA.

In mammals, nitrite toxicity presents primarily as methemoglobinemia. Typically, mammals are exposed to nitrate-contaminated food or water. The nitrate is converted to nitrite which oxidizes ferrous ion in hemoglobin to the ferric state which is incapable of transporting oxygen. Toxicity results from oxygen deprivation of cells. We propose a similar mechanism of acute toxicity of nitrite to daphnids. However, *D*. *magna* possess at least 7 hemoglobin-producing genes^27^ and are capable of rapid and significant modulation of hemoglobin levels^15^ in order to meet the oxygen requirements of the organism^28^. Here, we demonstrate that these variations in hemoglobin levels can affect tolerance of the organisms to the toxicity of nitrite.

Hemoglobin levels in daphnids are induced by at least two different pathways. The hypoxia signaling pathway results in increased hemoglobin production in response to low oxygen concentrations^29^. The methyl farnesoate signaling pathway increases hemoglobin production in response to other environmental cues such as photoperiod and temperature^30^. We have observed that some insect growth-regulating insecticides stimulate hemoglobin induction via the methyl farnesoate signaling pathway^21,31,32^. Pyriproxyfen, used in this study to elevate hemoglobin, is one such insecticide.

Human-generated hypoxia in aquatic habitats has become a major threat to the environment. Overuse of nitrogen-based fertilizers has resulted in the global occurrence of eutrophication and a growing number of hypoxic “dead” zones, now numbering in excess of 400^33^. Biota within these zones typically relocate or die^34^. Hypoxia is primarily responsible for the demise of inhabitants of these regions. In contrast, eutrophication at a level that does not induce lethally hypoxic condition tends to increase productivity at higher trophic levels^35,36^. Conceivably, the induction of hemoglobin at these margins of hypoxia may confer tolerance of some species to the toxic effects of nitrate and nitrite in these regions. Blue crab (*Callinectes sapidus*) and various other crustacean species have been shown to be tolerant of low oxygen conditions as they are capable of regulating oxygen transport^37^ presumably due to induction of oxygen-transporting proteins. Fish have been shown to increase hemoglobin levels in response to low oxygen conditions^38^.

Elevated temperatures can increase hemoglobin in aquatic species presumably due to the increasing oxygen demand of the organism coupled with the reduced oxygen solubility in water with increasing temperature. Further, increasing temperatures associated with climate change has been linked to an increase in the flux of nitrogen oxides into the environment^39^. Increased hemoglobin levels associated with climate change may prove to be protective against the commensurate accumulation of nitrate and nitrite in the environment.

## Materials and Methods

### Daphnia magna

*D*. *magna* used in these experiments have been cultured in our lab at North Carolina State University for over 20 years under conditions suitable for parthenogenetic reproduction as described previously^21^. In culture, daphnids were fed 2 mL (1.4 × 10^7^ cells) of green algae (*Pseudokirchneriella subcapitata*) and 1 mL (~4 mg dry weight) of Tetrafin® (Tetra, Spectrum Brands, Blacksburg, VA) fish food suspension twice daily. Preparation of the fish food suspension is described elsewhere^40^. During experiments, daphnids were fed 3.5 × 10^5^ cells of green algae once daily without fish food suspension.

### dsRNA

**Figure 6:**
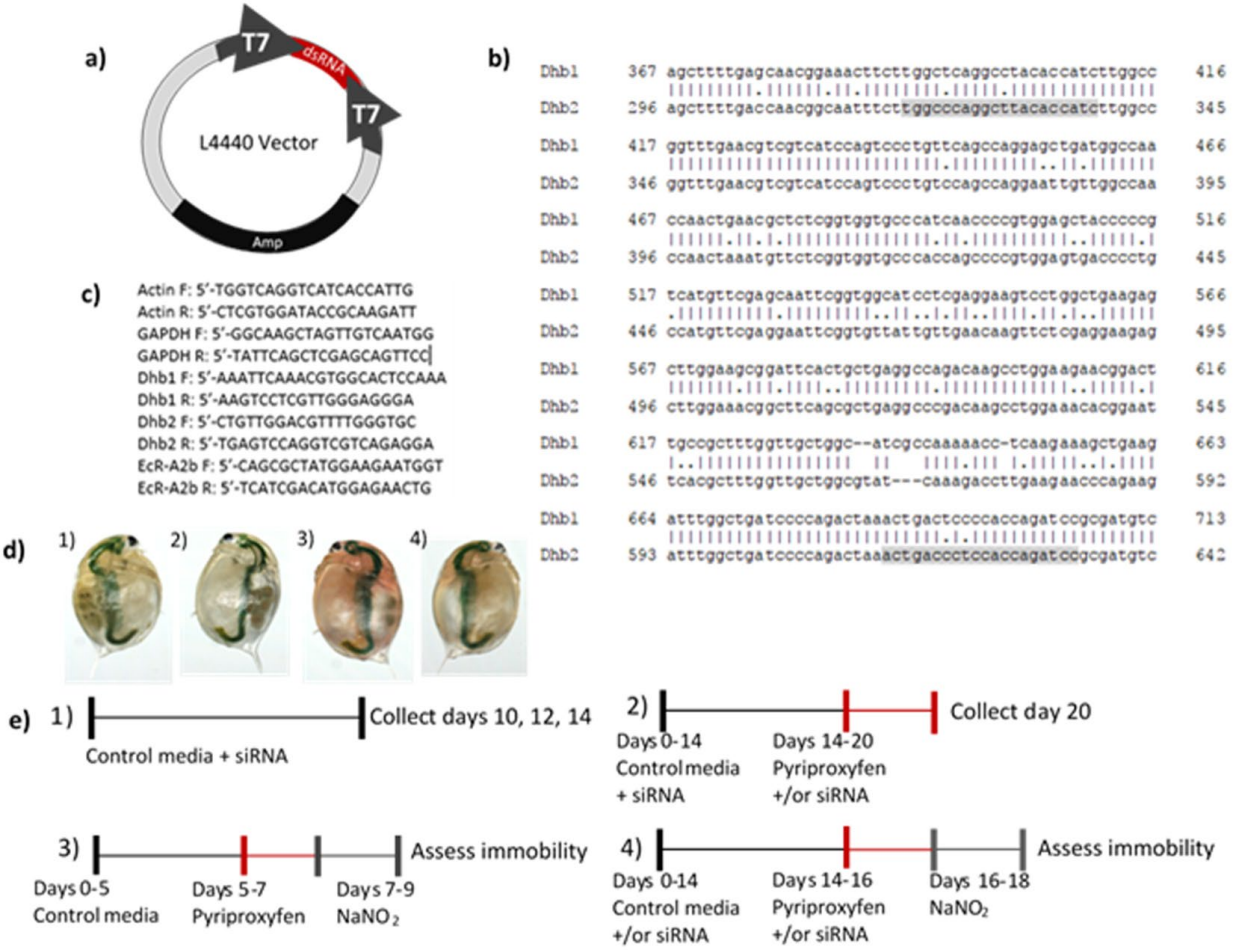
Methodological details. (**a**) Bacteria were transformed with the L4440 vector with or without the dsRNA target sequence. Location of the target sequence is indicated in red between two T7 promoters. The black region designates the ampicillin resistance gene. (**b**) The nucleotide sequence cloned into the L4440 vector for the production of dsRNA. Primers designed to clone the target sequence of Dhb2 are shaded in gray. These primers also amplified the paralogous sequence for Dhb1. (**c**) Primer sequences used for quantitative RT-PCR. (**d**) Coloration of daphnids reared in: 1) control media and fed bacteria containing empty L4440 vector, 2) control media and fed bacteria containing siRNA-containing L4440 vector, 3) media containing 6.0 nM pyriproxyfen and fed bacteria with empty L4440 vector, 4) media containing 6.0 nM pyriproxyfen and fed bacteria with siRNA-containing L4440 vector. (**e**) Treatment regimens used: 1) days of feeding siRNA required to knockdown targeted gene, 2) modulation of hemoglobin levels by exposure to pyriproxyfen and feeding siRNA, 3) sodium nitrite concentration-response assessment, and 4) toxicity of nitrite with modulated hemoglobin levels.

The L4440 vector, a gift from Dr. Andrew Fire (Addgene plasmid #1654), contains two T7 promoters which can be induced by isopropyl β-D-1-thiogalactopyranoside (IPTG) to produce dsRNA of the sequence ligated between these promoters (Fig. 6a). Primers were designed to clone a target sequence of 315 base pairs chosen from the nucleotide sequence of *D*. *magna* hemoglobin Dhb2 (Fig. 6b) and were prepared with the XbaI and KpnI restriction enzyme sites. The primers were synthesized by Integrated DNA Technologies, Inc (Coralville, IA). The desired sequence with the restriction enzyme sites was amplified according to the Phusion Hot Start II High-Fidelity PCR Master Mix protocol (ThermoFisher Scientific, Waltham, MA). The amplified target and L4440 plasmid vector were then double digested and ligated. The L4440 constructs were transformed into GC5 cells and plated on LB plates containing ampicillin (100 μg/mL, Sigma-Aldrich Corp., St. Louis, MO) and tetracycline (12.5 μg/mL, Sigma-Aldrich). Individual colonies were selected from LB plates and PCR was performed to assess whether ligation of the insert into the L4440 vector was successful. Successfully ligated vectors were sequenced (Eton Bioscience, San Diego, CA). After sequencing, the L4440 constructs were transformed into competent HT115 (DE3) cells obtained from the *Caenorhabditis* Genetics Center (funded by NIH Office of Research Infrastructure Programs (P40 OD010440)). Following transformation of HT115 cells containing the constructs, the bacterial cells were grown to OD_595_ = 0.04 (2.8 × 10^8^ cells/mL), and frozen at −80 °C. A dilution series of cells was prepared, and the optical density of each dilution at 595 nm was measured and used to generate a standard curve against cell count.

For experiments, transformed cells were gown overnight in LB medium containing ampicillin and tetracycline. IPTG (Sigma-Aldrich) was added at a concentration of 2 mM to induce production of the T7 RNA polymerase and subsequent production of dsRNA of the target sequence. At the time of feeding, optical density of the culture at 595 nm was measured and used to determine the number of cells per milliliter. Cells were centrifuged for 10 minutes at 3000 rpm and room temperature. LB medium was decanted and cells were resuspended in daphnid medium for feeding.

### mRNA Analyses

At the end of experiments, 3–4 daphnids per treatment were stored in 100 μL RNAlater (Invitrogen, Carlsbad, CA) in 1.7 mL tubes. Samples were stored at 4 °C for 24 hours then transferred to −80 °C until RNA extraction. Daphnids were homogenized using a Bullet Blender (Next Advance, Inc, Troy, NY), and RNA was isolated using the SV Total RNA Isolation System (Promega, Madison, WI). RNA concentration was determined by measuring absorbance at 260 nm using Nanodrop-1000 spectrophotometer (ThermoFisher). cDNA was prepared from extracted RNA using ImProm-II™ Reverse Transcription System (Promega) with oligo (dT) primers.

mRNA levels were measured by quantitative RT-PCR. Preparation of primers for *D*. *magna* actin, gapdh, Dhb1, Dhb2, and EcR-A (Fig. 6c) have been described previously^32,41^. Actin and gapdh were used to normalize Dhb1, Dhb2, and EcR-A mRNA levels. PCR was performed using the ABI PRISM ® 7000 Sequence Detection System with SYBR®Green PCR Mastermix (ThermoFisher) or iTaq Universal Sybr Green Supermix (BioRad, Hercules, CA) in 96-well plates (Olympus Plastics, Genesee Scientific, San Diego, CA) sealed with ThermalSeal (Excel Scientific, Inc., Victorville, CA). mRNA levels were calculated from raw data using Genex software from BioRad which utilizes algorithms to normalize mRNA levels to multiple housekeeping genes^42^.

### Optimization of hemoglobin suppression using siRNA

The siRNA methodology used in the study was based upon that described by Schumpert, *et al*.^22^. The optimal concentration of dsRNA-expressing bacterial cells required to effectively knock down expression of hemoglobin in daphnids consuming the cells was determined. Daphnids were provided 2.4 × 10^7^, 7.2 × 10^7^, or 12 × 10^7^ bacterial cells (per 100 mL medium) containing either empty vector or vector expressing Dhb2 dsRNA. At day 10, organisms were exposed to 3.0 nM pyriproxyfen (Sigma-Aldrich). Feeding of bacteria and exposure to pyriproxyfen continued until copper-coloration was observed among daphnids exposed to pyriproxyfen and provided empty vector (Fig. 6d). Daphnids were then collected and stored in 100 μL RNAlater at 4 °C for 24 hours and then moved to −80 °C until RNA was extracted.

### Hemoglobin modulation

Hemoglobin levels were elevated by exposing daphnids to 6.0 nM of the insecticide pyriproxyfen^31^. Pyriproxyfen was dissolved in 100% ethanol for delivery into the exposure solutions. The concentration of ethanol in all exposure solutions, including control was 0.05%. Hemoglobin levels were suppressed by exposing daphnids to bacteria containing Dhb2 dsRNA. Daphnids consume the bacteria resulting in the distribution of the siRNA throughout the organisms^22^. Exposure regimens for the various experiments are depicted in Fig. 6e. All exposures were conducted with animals <24 hours old, unless stated otherwise, in 40 mL medium in 50 mL beakers. Experiments were conducted in 16:8 hour light: dark cycle at 20 °C. Medium was changed every other day. Animals were fed 50 μL (3.5 × 10^5^ cells) green algae and 7.2 × 10^5^ cells/mL bacteria daily.

### Nitrite Toxicity

The impact of hemoglobin levels on the toxicity of nitrite was evaluated. First, the toxicity of nitrite to daphnids in which hemoglobin levels were not modulated was evaluated. Daphnids (7 days old) were exposed to concentrations of sodium nitrite ranging from 0–83 mg N/L for 48 hours after which immobility was determined. A daphnid was deemed immobile if it was on the bottom of the vessel and did not move for five seconds upon placing a pipet tip adjacent to it. Each treatment consisted of 10 daphnids isolated in 50 mL beakers containing 40 mL daphnid media. The experiment was conducted in 16:8 hour light: dark cycle at 20 °C. Similar experiments were performed concurrently where daphnids were exposed to 3.0 nM pyriproxyfen for 48 hours prior to initiating the exposure to sodium nitrite (Fig. 6e-3). EC50 values were calculated by preparing a non-linear fit of log-dose vs response in GraphPad Prism.

Subsequent experiments evaluating the toxicity of nitrite to daphnids pre-exposed to siRNA and/or pyriproxyfen were performed at a nitrite concentration of 25 mg N/L using methods as described above and the exposure regimen depicted in Fig. 6e-4. Each treatment consisted of nine organisms with two replicates. Results were analyzed by One-way Analysis of Variance with Tukey’s Multiple Comparisons Test using GraphPad Prism. Homogeneity of variances was confirmed with the Brown-Forsythe test.

### Data availability

The datasets generated during and/or analyzed during the current study are available from the corresponding author on reasonable request.
